# Effectiveness of controlling COVID-19 epidemic by implementing soft lockdown policy and extensive community screening in Taiwan

**DOI:** 10.1038/s41598-022-16011-x

**Published:** 2022-07-14

**Authors:** Ta-Chien Chan, Ching-Chi Chou, Yi-Chi Chu, Jia-Hong Tang, Li-Chi Chen, Hsien-Ho Lin, Kevin J. Chen, Ran-Chou Chen

**Affiliations:** 1 Research Center for Humanities and Social Sciences, Academia Sinica, 128 Academia Road, Section 2, Nankang, Taipei, Taiwan, ROC; 2 School of Medicine, Institute of Public Health, National Yang Ming Chiao Tung University, Taipei, Taiwan, ROC; 3 Department of Geography, National Taiwan University, Taipei, Taiwan, ROC; 4 Institute of Epidemiology and Preventive Medicine, National Taiwan University College of Public Health, Taipei, Taiwan, ROC; 5 Global Health Program, National Taiwan University College of Public Health, Taipei, Taiwan, ROC; 6 Department of Health, Taipei City Government, Taipei, Taiwan, ROC; 7 Department of Health, New Taipei City Government, 192-1, Yingshi Rd., Banqiao District, New Taipei City, Taiwan, ROC; 8 Department of Biomedical Imaging and Radiological Sciences, National Yang Ming Chiao Tung University, Taipei, Taiwan, ROC

**Keywords:** Epidemiology, Infectious diseases

## Abstract

Strict and repeated lockdowns have caused public fatigue regarding policy compliance and had a large impact on several countries’ economies. We aimed to evaluate the effectiveness of a soft lockdown policy and the strategy of active community screening for controlling COVID-19 in Taiwan. We used village-based daily confirmed COVID-19 statistics in Taipei City and New Taipei City, between May 2, 2021, and July 17, 2021. The temporal Gi* statistic was used to compute the spatiotemporal hotspots. Simple linear regression was used to evaluate the trend of the epidemic, positivity rate from community screening, and mobility changes in COVID-19 cases and incidence before and after a level three alert in both cities. We used a Bayesian hierarchical zero-inflated Poisson model to estimate the daily infection risk. The cities accounted for 11,403 (81.17%) of 14,048 locally confirmed cases. The mean effective reproduction number (Re) surged before the level three alert and peaked on May 16, 2021, the day after the level three alert in Taipei City (Re = 3.66) and New Taipei City (Re = 3.37). Mobility reduction and a lower positive rate were positively associated with a lower number of cases and incidence. In the spatiotemporal view, seven major districts were identified with a radial spreading pattern from one hard-hit district. Villages with a higher inflow degree centrality among people aged ≥ 60 years, having confirmed cases, specific land-use types, and with a higher aging index had higher infection risks than other villages. Early soft lockdown policy and detection of infected patients showed an effective strategy to control COVID-19 in Taiwan.

## Introduction

From January 2020, the COVID-19 pandemic has swept over the world, causing high morbidity and mortality^1,2^. In 2020, most countries had non-pharmaceutical approaches to slow down the epidemic, such as lockdowns^3^, social distancing^4^, mask-wearing^5^, and controlling international borders^6^. However, the strict and repeated lockdown caused resident fatigue on policy compliance and had a large impact on economies^7^. At the early stage of the pandemic, a social distancing policy was supported by residents and this significantly reduced the spread of severe acute respiratory coronavirus 2 (SARS-CoV-2), the causative agent of COVID-19, worldwide^8^. Nevertheless, the high transmissibility and high mutation rate of SARS-CoV-2 variants continue to pose global public health threats^9^. In 2021, enhancing vaccination coverage to cope with the pandemic became a major public health priority in countries.

Given that COVID-19 spread through contact between humans, a full lockdown, including restricted social contact and keeping open essential businesses only, places everyone in quarantine and minimizes the risk of community transmission. Evidence showed that a 2-week lockdown successfully reduced the number of daily diagnosed cases, intensive care unit admissions, and mortality in Italy and Spain^10^. Nonetheless, Taiwan never enforced strict lockdowns, nor did it resort to drastic restrictions on civil freedom. Instead, a more lenient approach was adopted, encouraging residents to stay at home and follow protocols, including mask-wearing and limits on gatherings. Since Taiwan has not reached the level four alert criteria (a daily average of above 100 cases over the last 14 days with at least half transmitted from unknown sources), a full lockdown was unnecessary. The soft lockdown measures were more sustainable as people voluntarily stayed at home and adhered to the regulations.

In Taiwan, the government has also implemented significant efforts to implement border control, quarantine policy, contact tracing, social distancing policy^11^, and personal protective behavior, including the wearing of masks and alcohol handwashing^12^. These policies successfully suppressed the local COVID-19 epidemic from January 2020 to April 2021. On May 15, 2021, the Central Epidemic Command Center (CECC) of Taiwan declared a level three alert in Taipei City and New Taipei City because of community outbreaks. On May 19, 2021, CECC issued a nationwide level three alert and downgraded to level two alert on July 27, 2021. During the level three alert period, there were no strict regulations on the suspension of work; however, all schools were closed. People had to wear masks at all times, and indoor gatherings were limited to five people, while outdoor gatherings were restricted to 10^13^. A wide spectrum of specific businesses and public venues were to be closed, except for essential services, such as police departments, hospitals, and government buildings. In those businesses and public institutions that remained open, crowd control, masks, and social distancing were required. Workplaces had to abide by epidemic prevention requirements, implement personal and workplace hygiene management, and initiate continuous corporate operation response measures, such as remote work and flexible working hours. Restaurants and coffee shops could only provide take-out services. In contrast to the “hard” lockdown in other countries where there was restricted freedom of movement^14,15^, the residents in Taiwan were not restricted to move but were encouraged to stay at home. Therefore, we named this type of level three alert as “soft” lockdown.

In addition, the campaign for extensive polymerase chain reaction (PCR) testing in the communities was launched in mid-May 2021, the early phase of this outbreak. The local governments in Taipei and New Taipei City encouraged residents who lived close to the hotspot areas or had any influenza-like symptoms (before June 2021; while residents without any symptoms could also receive the test after June 2021) to undergo a PCR testing at community screening stations.

With a soft lockdown policy and extensive rapid screening, the effectiveness of controlling the COVID-19 outbreak within 2 months was to be evaluated. To balance socio-economic development and disease control, timely and precise intervention was needed to lower the socioeconomic impact and flatten the curve of the epidemic. The study area was the two metropolitan cities of Taipei City and New Taipei City in northern Taiwan with 6.44 million residents, accounting for 27.78% of the total Taiwanese population, which also represented the political and economic centers of the country. The wave in mid-2021 started in these two cities, accounting for 81.17% of the total confirmed COVID-19 cases at the time. This study was based on epidemic, mobility, and land-use data in Taipei and New Taipei City, as well as information on policy interventions, to evaluate the policy effect on COVID-19 at different spatial resolutions.

## Results

**Figure 1:**
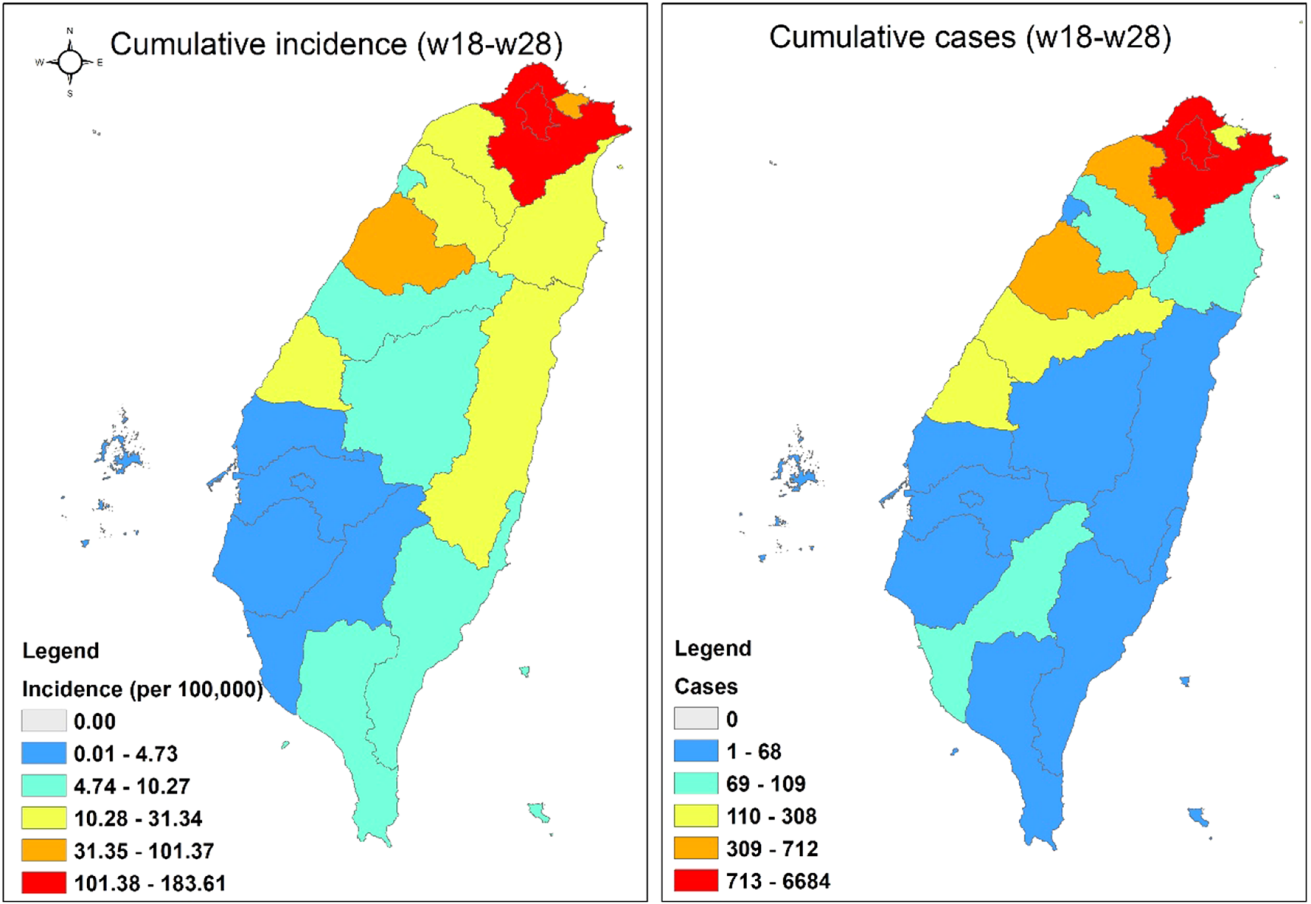
Cumulative incidence and cases of indigenous COVID-19 cases from week 18 (May 2 to May 8) to week 28 (July 11 to July 17, 2021). The map was created by ArcGIS (ArcMap, version10.3; ESRI Inc., Redlands, CA, USA).

During our study period (from weeks 18 to 28 in 2021), the cumulative incidence and cases were high and clustered in northern Taiwan (Fig. 1), especially in Taipei City (4719/14,048, 33.59%) and New Taipei City (6684/14,048, 47.58%). The two cities accounted for 11,403 cases (81.17%) of the total 14,048 local confirmed cases in these 11 weeks.

**Figure 2:**
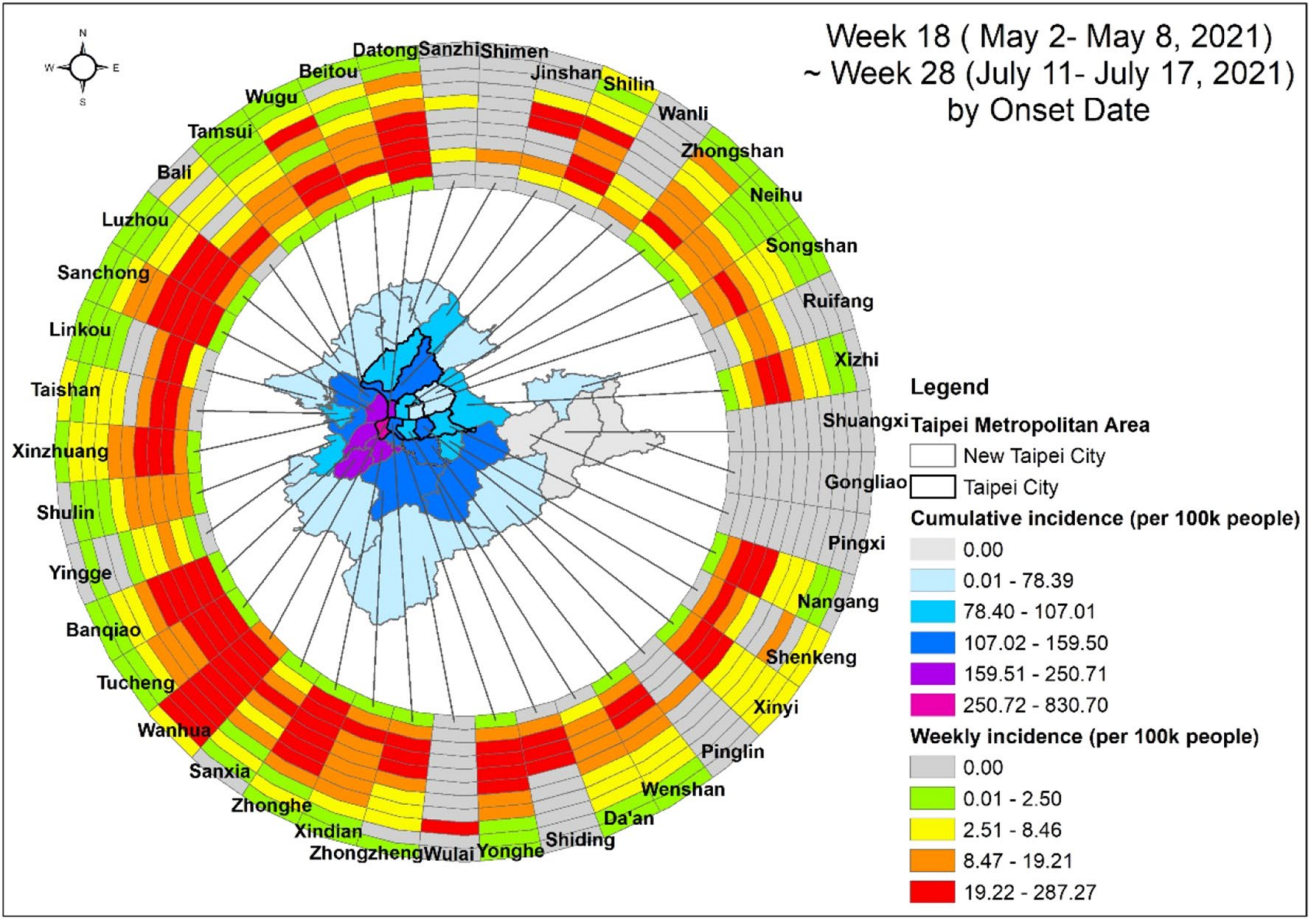
Spatiotemporal dynamics of COVID-19 incidence at the district level in Taipei and New Taipei City from week 18 to week 28, 2021. The map was created by ArcGIS (ArcMap, version10.3; ESRI Inc., Redlands, CA, USA) and the ring map was created by the ring map toolbox in ArcGIS (https://www.esri.com/about/newsroom/arcuser/looking-at-temporal-changes).

We explored the spatiotemporal dynamics of COVID-19 incidence for this major outbreak area using a ring map (Fig. 2). In the middle of the map, we show the cumulative incidence among these 11 weeks. The Wanhua district of Taipei City had the highest incidence. The neighboring districts around the Wanhua district in purple had the second-highest cumulative incidence. Subsequently, the third and fourth waves were away from the hardest-hit district, Wanhua, and the incidence declined radially from Wanhua. In the ring map, the innermost ring is week 18 (from May 2 to May 8), and the outermost ring is week 28 (from July 11 to July 17). Wanhua had an earlier strong signal at week 18, and the neighboring districts also had some sporadic signals. The weekly incidence was high at week 19. Taipei City and New Taipei City had level three alerts on May 15, the last day of week 19. In week 20, the third ring of the ring map had a very high incidence. Some districts peaked for two to three weeks before showing a decline. However, some districts peaked for over 5 weeks.

**Figure 3:**
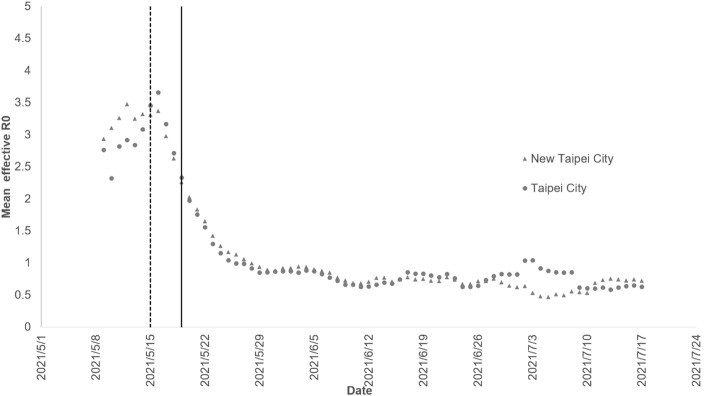
Daily mean effective reproduction number in Taipei and New Taipei City. The left vertical dashed line is the level three alert in Taipei and New Taipei City on May 15, 2021. The right vertical solid line is the level three alert throughout Taiwan on May 19, 2021.

The 7-day window of mean effective reproduction number surged before the level three alert and peaked on the day after the level three alert in Taipei City (May 16, 2021, R0: 3.66) and New Taipei City (R0: 3.37) (Fig. 3). At the end of May, the effective R0 declined to < 1 after 2 weeks of level three alert. Between the end of June and the beginning of July, there was one cluster transmission in the markets and the effective R0 increased slightly during that period.

**Figure 4:**
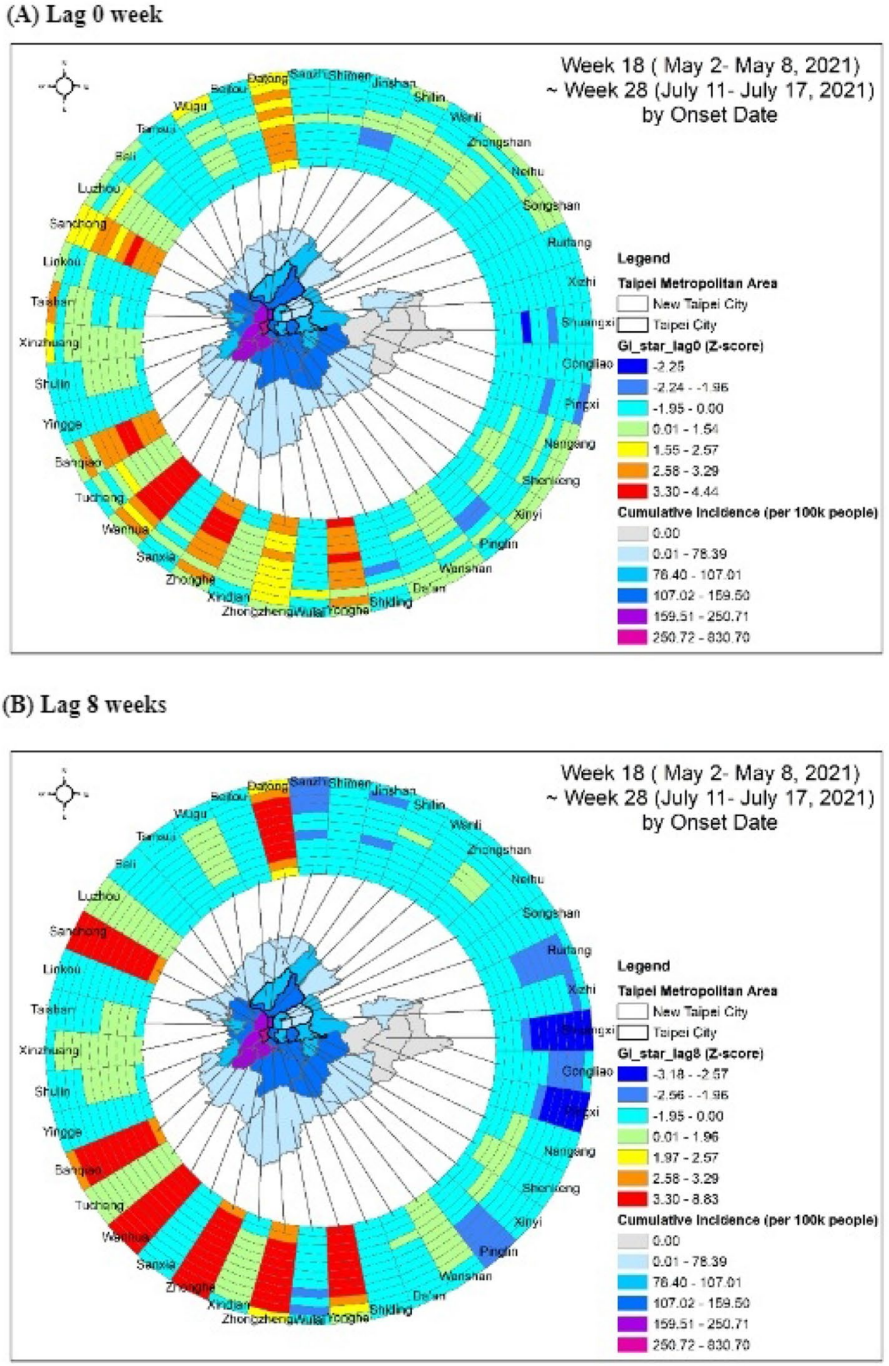
Spatiotemporal hotspot detection of COVID-19 incidence at the district level in Taipei and New Taipei City. The map was created by ArcGIS (ArcMap, version10.3; ESRI Inc., Redlands, CA, USA) and the ring map was created by the ring map toolbox in ArcGIS (https://www.esri.com/about/newsroom/arcuser/looking-at-temporal-changes).

The spatiotemporal autocorrelation was considered to determine the persistent hotspots (Fig. 4). In Fig. 4A, seven purely spatial hotspots were found each week. They were the Datong, Wanhua, and Zhongzheng districts in Taipei City and the Sanchong, Banqiao, Zhonghe, and Yonghe districts in New Taipei City. After considering the maximum 8 weeks of temporal dependence, the seven hotspots clearly showed persistent effects on the COVID-19 epidemic (Fig. 4B).

To measure the effectiveness of extensive screening in communities and mobility changes in the COVID-19 epidemic, we stratified the analyses into two periods: one before the level three alert was issued (May 2 to May 14) and the other after this alert (May 17 to July 17, Table 1). The second period aimed to measure the effect of the positivity rate. Thus, we used the intersection time period of the positive rate in both cities. In the first period, the cases and incidence increased with time and were high on the weekend. The association between mobility and cases/incidence was negative but not significant in cases. In the second period, negative associations between cases/incidence and time were noted (*p* < 0.001). Positive associations between cases (*p* = 0.69)/incidence (*p* < 0.001) and positive rate and between cases (*p* < 0.001)/incidence (*p* < 0.001) and mobility were observed.

**Table 1:** The policy effect on the daily COVID-19 cases and incidence at city level.

Variables	5/2–5/14 (before level three alert)				5/17–7/17 (after level three alert)			
	Cases		Incidence		Cases		Incidence	
	Coefficient (SD)	P	Coefficient (SD)	P	Coefficient (SD)	P	Coefficient (SD)	P
Intercept	38.9 (37.5)	0.311	6.10 (2.46)	0.021	133.9 (14.8)	< 0.001	8.47 (0.85)	< 0.001
Linear temporal trend	7.10 (1.58)	< 0.001	0.45 (0.10)	< 0.001	− 3.48 (0.22)	< 0.001	− 0.19 (0.01)	< 0.001
Positive rate	–	–	–	–	0.79 (1.98)	0.69	0.59 (0.11)	< 0.001
Weekend	17.4 (11.6)	0.149	1.79 (0.76)	0.028	− 9.30 (5.84)	–	–	0.011
Apple mobility on transit	− 0.82 (0.43)	0.066	− 0.10(0.03)	0.002	1.91 (0.39)	< 0.001	0.08 (0.02)	< 0.001

**Figure 5:**
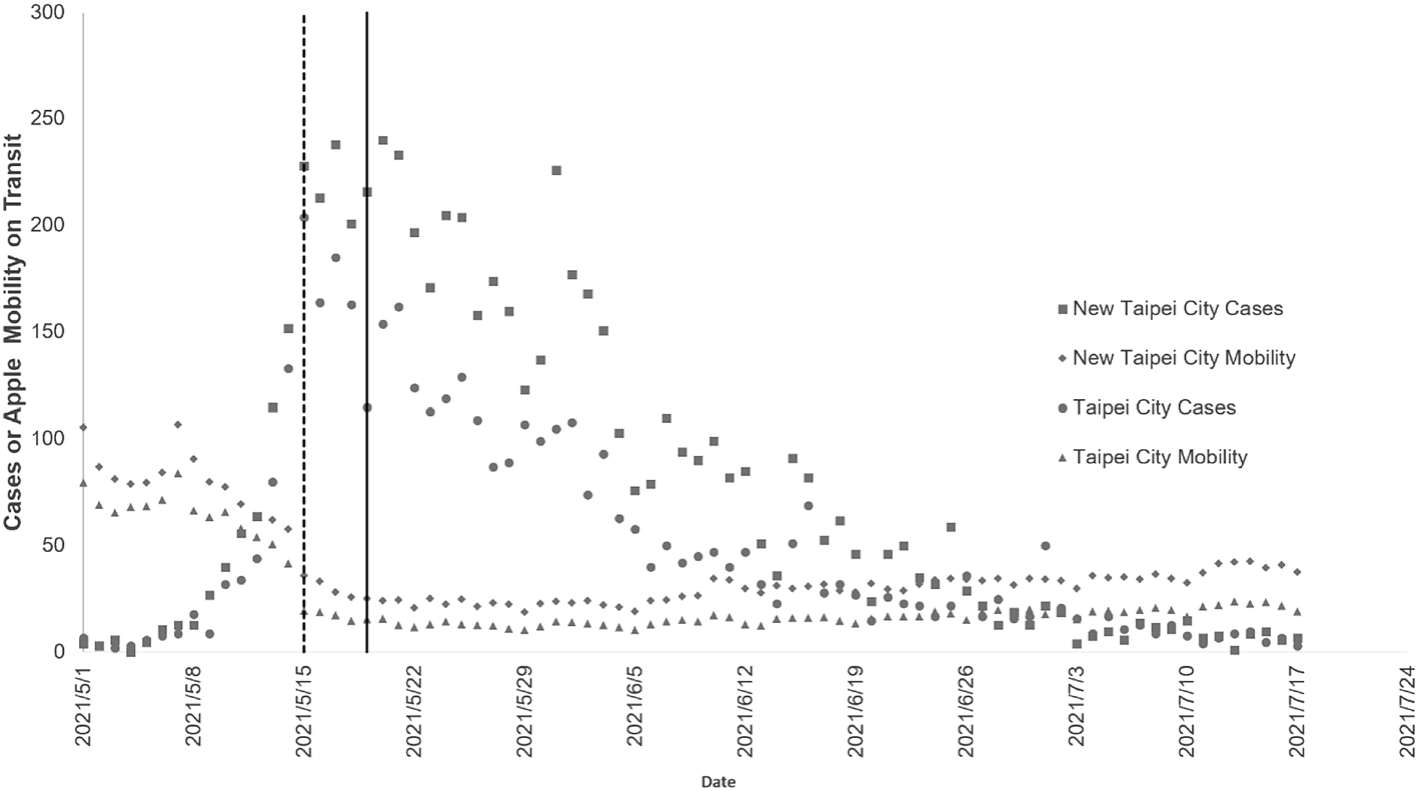
Association between daily COVID-19 cases and Apple mobility indicator on transit in Taipei and New Taipei City. The left vertical dashed line is the level three alert in Taipei and New Taipei City on May 15, 2021. The right vertical solid line is the level three alert throughout Taiwan on May 19, 2021.

 In Fig. 5, a similar temporal pattern was observed in the effective R0 pattern. The number of cases declined after the level three alert. The mobility data by transit from Apple mobility trends reports showed a significant reduction after the level three alert in both cities.

**Figure 6:**
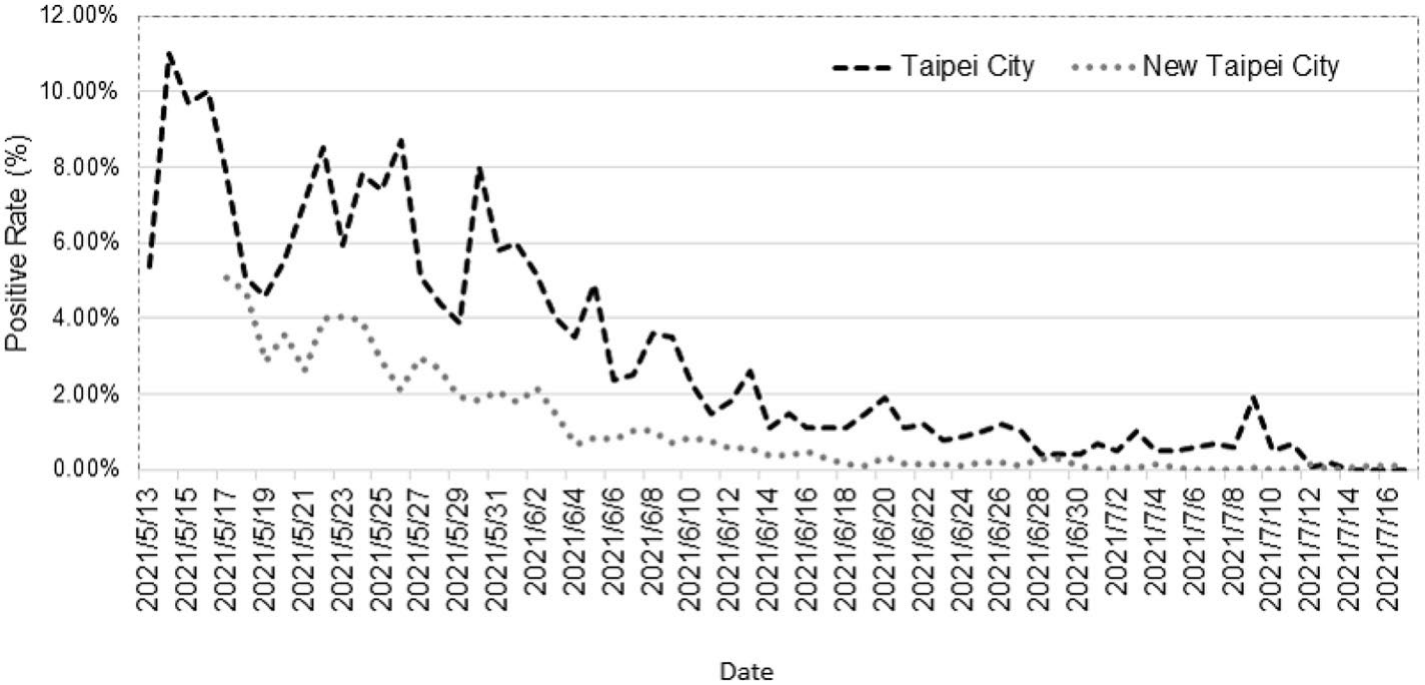
Temporal trend of COVID-19 positive rate by polymerase chain reaction (PCR) testing in the communities.

COVID-19 positive rates from the rapid screening station started in mid-May 2021 (Fig. 6). The infected cases at the beginning of the outbreak were mostly from Wanhua District, Taipei City. The Taipei City and New Taipei City governments established a rapid screening station at the early stage of the outbreak. The absolute positive rate was affected by the number of screened people and the source of the population located in hotspot areas. The temporal trend of the positive rate reflected the decrease in COVID-19 transmission in the communities. At the end of the study period, the positive rates were close to zero in both cities.

We estimated the effects of predictors on daily confirmed COVID-19 cases at the village level based on the Bayesian hierarchical zero-inflated Poisson model (Table 2). The posterior means effect with 95% credible intervals ranging from 0.033 to 0.052 showed that if the villages had more confirmed cases in the previous 3 days, the corresponding villages would have more confirmed cases today.

**Table 2:** The effects of mobility structure changes and types of land utilization on daily COVID-19 cases at the village level.

Variables	Posterior mean	Std. dev.	95% credible interval	
			Lower	Upper
(Intercept)	− 10.556	0.176	− 10.904	− 10.214
The cumulative cases from the previous 3 days	0.042	0.005	0.033	0.052
**In-degree centrality**				
18–21	− 0.008	0.009	− 0.026	0.007
22–29	− 0.006	0.003	− 0.013	0.001
30–59	− 0.001	0.001	− 0.002	0.001
≥ 60	0.011	0.002	0.007	0.016
**Out-degree centrality**				
18–21	0.008	0.005	− 0.002	0.017
22–29	0.000	0.003	− 0.006	0.006
30–59	0.000	0.001	− 0.001	0.002
≥ 60	− 0.007	0.003	− 0.012	− 0.002
**Percentage of different land utilization within the village**				
Government agencies	− 2.104	1.035	− 4.226	− 0.161
School	0.676	0.423	− 0.162	1.499
Medical facilities	− 4.770	2.459	− 9.769	− 0.110
Social welfare facilities	2.837	1.196	0.378	5.074
Parks and greenspace	0.263	0.519	− 0.764	1.275
Commercial	1.712	0.677	0.374	3.034
Residential	− 1.023	0.428	− 1.865	− 0.184
Mixed-use housing	1.137	0.483	0.187	2.084
Manufacturing areas	1.068	0.841	− 0.605	2.698
**Population density**				
Group 1 (< 5984.18)	The reference group			
Group 2 (5984.18 to 24,858.65)	0.191	0.145	− 0.092	0.476
Group 3 (24,858.65 to 44,493.69)	− 0.025	0.193	− 0.403	0.354
Group 4 (≥ 44,493.69)	0.173	0.234	− 0.287	0.633
**Aging index**				
Group 1 (< 119.56)	The reference group			
Group 2 (119.56 to 165.88)	0.364	0.092	0.183	0.545
Group 3 (165.88 to 219.78)	0.382	0.096	0.194	0.571
Group 4 (≥ 219.78)	0.508	0.115	0.282	0.734

Interestingly, we also found that the structure of mobility among villages played a role in the increasing number of confirmed cases. Villages with a higher inflow degree centrality among people aged ≥ 60 years had a significant positive association with the increase in confirmed cases than other villages (95% credible interval, 0.007 to 0.016). On the contrary, villages with a higher number of outflow degree centrality among people aged ≥ 60 years had a significant negative association with the increase in confirmed cases than other villages (95% credible interval, − 0.012 to − 0.002).

In addition to the mobility effect, villages composed of different land-use types may have different infection risks. We found that there were three major types of land use associated with the increase in confirmed cases in this outbreak (social welfare facilities, commercial, and mixed-use housing) and three with their decrease (government agencies, medical facilities, and purely residential areas). Population density and aging index were equal-frequency discretization into four groups: Group 1 (< Q_1_), Group 2 (Q_1_ to Q_2_), Group 3 (Q_2_ to Q_3_), and Group 4 (≥ Q_3_), where Group 1 of the two predictor variables was used as the reference group. The model parameter estimates revealed that the four population density groups had no significant effect on the daily number of confirmed cases. Furthermore, positive mean effect differences between the other three groups and the reference one were observed in aging index groups. This indicated that the number of confirmed cases increased with an increased aging index.

## Discussion

Lockdown policy increasingly appears to be necessary for governments to slow the pandemic if community transmission outbreaks occurred before or during the vaccination campaign^16^. Hence, it is crucial to provide statistical evidence of the effect of lockdown measures and the extent to which mobility influences disease transmission. This study provided data-driven evidence on the positive impact of the Taiwan soft lockdown measures on COVID-19 at a high spatiotemporal resolution. Specifically, the incorporation of mobility data at the village level facilitates dynamic intervention monitoring and disease surveillance.

Regarding the mobility restriction policy, a strong positive association between cases/incidence and mobility was observed after level three alert. This was in agreement with previous findings showing that for 52 countries that have experienced or continue to experience active SARS-CoV-2 transmission, there was a strong link between mobility measures and transmissibility^17^. This relationship is important for the optimization of the mobility restriction policy. Thus, aggregated mobility data play a critical role in optimizing interventions by providing real-time information.

In addition to a soft lockdown policy in Taiwan, literature has highlighted that the digital resilience of the healthcare system and the support from the primary care system could alleviate the hospital capacity and reduce the severity of COVID-19 infection. Digital transformation of teleconsultation was rapidly adopted in the healthcare setting during the pandemic period. One study conducted a survey in France to elucidate the acceptance and trust of teleconsultation from the patients, which might play a role in curtailing disease transmission and facilitating treatment-seeking for suspected or mild COVID-19 symptoms^18^. Another study in Greece proposed that augmentation of the healthcare system by reinforcing primary and community care could prove to be a lasting disease containment strategy^19^. During the mid-2021 COVID-19 outbreak in Taiwan, the infected cases mostly occurred in the two cities of northern Taiwan where medical resources were abundant. Although the infected cases quickly surged in the 2 weeks, the level three alert policy effectively reduced the transmission at that wave of the outbreak. Every resident, except for the infected confirmed cases, could move freely. The infected persons could choose to quarantine at home or at the collective quarantine facility. If they had any risk factors for developing severe cases, such as older age or comorbidities, they would be quarantined at the hospitals for healthcare. At that time, the number of infected people needing clinical treatment was still below the capacity of the hospitals. Therefore, digital teleconsultation and the strength of primary care, which do offer some advantages in other countries, were not used in the mid-2021 outbreak in Taiwan.

In addition, mass testing can also help suppress the spread of COVID-19, which was demonstrated by a modeling study on the clinical effectiveness of population-scale testing. One study projected that mass testing would reduce the basic reproductive number and mortality rate, and further cut the chain of transmission by using a susceptible-infected-recovered (SIR) model^20^. Followed by the large-scale rapid screening, the identification of local cases followed by meticulous contact tracing and stringent quarantine of close contacts were also crucial to containing SARS-CoV-2.

Our study provided spatiotemporal evidence that a soft lockdown accompanied by clear guidelines, public discipline, and extensive testing can serve as a disease-mitigating intervention to curb community transmission. Although the estimated effectiveness of public health interventions in Taiwan was previously described^11,21^, the focused approaches and the epidemic situation were entirely different in this study. The mentioned studies were conducted during a period of low numbers of cases in 2020 in Taiwan. They found that the two successful approaches to slow down the transmission and flatten the epidemic curve included case-based, such as case detection, contact tracing, and 14-day quarantining of close contacts, and population-based interventions, such as physical distancing and mask-wearing. However, this study focused on a large outbreak in 2021 which was the first COVID-19 epidemic affecting Taiwanese communities. When facing the surging number of infected cases, the soft lockdown strategy and extensive community screening were first introduced and bundled with individual and population-based interventions. We provided combinatorial evidence regarding aggregated mobility data and corresponding mean effective reproduction number to ascertain the efficacy of soft lockdown and mass testing after the local COVID-19 outbreak.

Wanhua District, which had the highest COVID-19 cumulative incidence, and its surrounding six districts were identified as significant hotspots, indicating the geospatial dynamics of the spread of the virus to peripheral districts and the persistent effects on the epidemic. A similar spatiotemporal pattern of COVID-19 was also seen in Bangladesh, indicating that cases were first confined within the central region of the country, Dhaka, and subsequently spread to the surrounding six districts, which were later also identified as significant hotspots^22^. These findings provide insights into the geo-statistical analysis of COVID-19 clusters, which may assist public health agencies in predicting spatiotemporal transmission dynamics and formulating control strategies.

According to the policy stringency index provided by the Oxford COVID-19 government response tracker^23^, the average stringency index of Taiwan before May 1, 2021, was 24.04 and the one during the level three alert period in 2021 was 70.29. Between May and July 2021, the number of countries, with an average monthly stringency index exceeding 70, was 27 worldwide. We chose five countries with similar stringency indexes for comparison: Germany (70.13), Canada (71.07), Vietnam (76.29), Argentina, 78.51, and India (80.44). The stringency index was composed of eight containment and closure policy indicators plus one indicator recording public information campaigns. The comparison of policy differences in these six countries is briefly listed in Supplementary Appendix 1.

Regarding school closures (C1), cancellation of public events (C3), restrictions on gatherings (C4), and public information campaigns (H1), six countries had a consistent policy on school closures, cancellation of public events, restrictions of gatherings involving ≤ 10 people, and had coordinated public information campaigns. For the rest of the five indicators in the stringency index, the six countries had different policy strengths, which related to human movement and social activity. Regarding workplace closures (C2), Taiwan only required the closure of some businesses, but other countries such as India and Canada require closing for all-but-essential workplaces. Closure of public transport (C5) was the major difference between Taiwan and other countries. There was no measure on closing public transport and residents were recommended to stay at home (C6) and not to travel between cities (C7) in Taiwan. Compared to other countries, they adopted required and recommended closures to reduce the human flow and social contacts. For this reason, we termed this policy in Taiwan a soft lockdown, as residents could adjust their behavior to adhere to the policy, while maintaining their flexibility and freedom to move. The last stringency index indicator was international travel (C8), which related to the imported risk from other countries. In Argentina, Canada, Taiwan, and Vietnam, strict border control was applied. In Germany and India, they banned arrivals only from some regions. In addition to the stringency index, the open testing policy (H2), comprehensive contact tracing (H3), and the mandatory facial covering outside the home (H6) were also successful strategies applied in Taiwan to combat the mid-2021 COVID-19 outbreak.

The findings of this study have unique implications for the combinatory analysis of land-use and demographic data, along with the COVID-19 epidemic in Taiwan. Interestingly, villages with a higher inflow population aged over 60 years were associated with higher infection risks compared to other villages. This could be explained by the Wanhua transmission chain, in which senior citizens accounted for a substantial part. Not only were age-related variables, that is, aging index and percentage of the population aged 65 years and older, proven to be significant to the spread of COVID-19 in a previous study^24^, but our study revealed that villages with a higher aging index were associated with a higher susceptibility to infection. This was also due to the deteriorating immune system and underlying medical problems that predispose them to more severe infections^25^. In addition to the mobility effect, villages with different land-use types had distinct impact on infection risks. We found that there were three major types with higher infection risk in this outbreak: social welfare facilities, commercial and mixed-use housing, which were associated with cluster infections in the long-term care facilities, and higher mobility of crowds. Such findings shed light on demographic and land-use heterogeneities associated with infection risk, which could enable precise disease prediction and prevention.

This study had some limitations. First, despite large-scale testing, an underestimation of incidence was expected because of undetected asymptomatic cases. However, the study has extended over a time span of 11 weeks (May 2 to July 17, 2021), in which asymptomatic carriage and transmission clusters could still be reflected in the cases and incidence we collected. In hindsight, given the downward epidemic trend in Taiwan, the impact of this underestimation was minimal. Second, with the ongoing COVID-19 pandemic, the data might change over time, but the spatiotemporal pattern of COVID-19 presented in this study could still serve as a vital reference for lockdown decision-making and aggregated mobility applications. Third, when we modeled the mobility data with different age groups on the villages’ incidences, we could not differentiate the age distribution of the confirmed cases in that village. However, infected patients might not only transmit the virus to the same age group but also to different age groups. Therefore, risk can be treated as the overall infection risk in the community. Fourth, the representativeness of the telecommunication data was not comprehensive. The aggregated mobility data we used was from FarEastern telecommunication company, which accounted for 23.86% of the market share in Taiwan. As we could not apply the data from other four companies, we could not ascertain the representativeness of all residents in these two cities.

In conclusion, the soft lockdown policy was considered to balance socio-economic activity and the mitigation of COVID-19 transmission. Despite extensive studies focused on the relationship between lockdown and mobility, there is a paucity of research regarding the effectiveness of COVID-19 outbreak control by a soft lockdown policy. An advantage of this study was the utilization of finer spatiotemporal disease surveillance data and estimation of the structure and quantity change of human mobility from telecommunication data. Capturing the spatiotemporal dynamics of epidemics could become a forecasting tool. During outbreaks, the evaluation of the effectiveness of policy interventions is important. In our findings, we found that a soft lockdown policy and extensive screening played a role in slowing down the transmission. The control measures used in this study aimed to allow society to operate normally, without forbidding the freedom of movement of the residents along with other public health interventions. This might provide an alternative to traditional lockdown measures in containing COVID-19 and preventing future epidemics.

## Methods

### Ethics

This study was approved by the Research Ethics Committee of the National Taiwan University (202007HM008). The data used in this study were all aggregated and anonymized, and thus the requirement for informed consent was waived by the Research Ethics Committee of the National Taiwan University. This study was performed in accordance with the declaration of Helsinki and followed by the approved protocol.

### Data

The confirmed COVID-19 cases and their age distribution at the township level are from open data maintained by the Taiwan Centers for Disease Control and Prevention (CDC) (https://data.gov.tw/dataset/120711). The confirmed COVID-19 cases at the village level were from the Department of Health in Taipei and New Taipei City. There are 456 villages in Taipei City and 1032 villages in New Taipei City. The study period was defined from May 2, 2021, to July 17, 2021. The positive rate of community PCR testing in Taipei and New Taipei City is from press releases. The expense for the test was entirely covered by the government. The mobility data at the village level were provided by the local commercial telecommunication company, Far EasTone Telecommunications. The land use data in 2019 and demographic data including population density and aging index in June 2021 at the village level were obtained from the socio-economic database maintained by the Ministry of Interior, Taiwan (https://segis.moi.gov.tw/STAT/Web/Portal/STAT_PortalHome.aspx). According to the land-use classification system in Taiwan (https://www.rootlaw.com.tw/LawContent.aspx?LawID=A040040100025100-1080328), there are nine categories in the first tier, 41 subcategories in the second tier, and 103 subcategories in the third tier. In this study, we subjectively selected 13 subcategories from the second tier into the model selection. Finally, we obtained nine types of land-use in the final model, including government agencies, schools, medical facilities, social welfare facilities, parks and green spaces, commercial, residential, mixed-use housing, and manufacturing areas.

#### Spatiotemporal trend of COVID-19 incidence and hotspot detection

 To visualize the spatiotemporal dynamics of the COVID-19 outbreak in Taipei and New Taipei City, we used ArcGIS (ArcMap, version10.3; ESRI Inc., Redlands, CA, USA) and our developed ring map toolbox (https://www.esri.com/about/newsroom/arcuser/looking-at-temporal-changes) to display the weekly incidence at the district level for 11 weeks. To detect the spatiotemporal hotspots of confirmed COVID-19 incidence, we applied a modified spatiotemporal Getis-Ord Gi* statistic^26^ to consider their spatiotemporal dependence. The detailed procedure is listed in Supplementary Appendix 2.

### Computing effective reproduction number

Following the meta-analysis results from Challen et al.^27^, we assumed a gamma distribution for the serial interval with a mean of 4.97 and a standard deviation of 4.23 days and calculated time-dependent daily reproduction numbers over a 7-day moving window. The time-dependent daily reproduction numbers were computed using R 3.6.3 (R Core Team)^28^ and the EpiEstim (v2.2-4) package^29^.

### Mobility data and social network indicator

The mobility data on transit in both cities were obtained from the Apple Mobility Report (https://covid19.apple.com/mobility). The mobile phone data used in this study were collected and aggregated by Far EasTone Telecommunications in Taiwan. During the study period, we obtained a three-day mobility flow matrix by four age groups (age groups: 18–21, 22–29, 30–59, ≥ 60 years) from 7753 villages. Three days included May 10 (before level three alert in Taipei and New Taipei City), May 16 (after level three alert in Taipei and New Taipei City), and May 20 (after level three alert across Taiwan). The observed population was defined as the users of FarEasTone telecommunication. The telecommunication company first defined their observed population by computing their mobile phone users aged ≥ 18 years old and staying in the villages overnight in the previous month. We computed the inflow and outflow weighted degree centrality by the mobile phone data as the measure of mobility structure change before and after the level three alert. The detailed computation method is listed in Supplementary Appendix 3.

### Statistical analysis

In the first part, simple linear regression was applied to evaluate the overall impact on the temporal trend of COVID-19 cases and incidence in both cities. We stratified the two periods for analysis, including one period before level three alert (May 2, 2021, to May 14, 2021) and the second period after level three alert (May 17, 2021, to July 17, 2021). The explanatory variables included linear temporal trend, positive rate from community screening, weekend effect, and Apple mobility data on transit.

In the second part, the Bayesian hierarchical zero-inflated Poisson model (type 0)^30^ with spatial and nonspatial structured random effects was constructed to estimate the effects in each village of Taipei and New Taipei City using R software^28^ and the package of INLA (www.r-inla.org)^31^.

The likelihood models combining spatially structured and unstructured effects of village *i* in Bayesian modelling is given by:

$${E(y}_{i})=\alpha +\mathrm{log}\left({s}_{i}\right)+{{\varvec{x}}}_{i}^{T}{\varvec{\delta}}+{u}_{i}+{v}_{i},$$

where ${y}_{i}$ is cases in village *i*, and $\mathrm{log}\left({s}_{i}\right)$ is the offset term, in which ${s}_{i}$ is the population of village *i*. ${u}_{i}$ and ${v}_{i}$ are the spatial structured and unstructured random effect of village *i*, respectively. The ${{\varvec{x}}}_{i}$ is region covariates of village *i*, including the cumulative cases from the previous 3 days, in-degree centrality and out-degree centrality by four age groups, and the percentage of nine types of land utilization within the village, population density, and aging index. The ${\varvec{\delta}}$ is regression parameter. For the prior distributions, ${u}_{i}$, ${v}_{i}$, and all regression coefficients were given independent prior normal distributions with zero mean and large variance ${\sigma }^{2}$ (or equivalently small precision), i.e. $N\left(0, {\sigma }^{2}\right)$ and we set ${\text{log}}\left({1}/{\sigma }^{2}\right)\text{ }{\text{log Gamma}}\, \left(\text{1, 0.0005}\right)$.

## Supplementary Information

Supplementary Information 1.

Supplementary Information 2.

Supplementary Information 3.

## Data Availability

The confirmed COVID-19 cases at the township level were from open data maintained by the Taiwan CDC (https://data.gov.tw/dataset/120711). The confirmed COVID-19 cases at the village level were from the Department of Health in Taipei and New Taipei City. The positive rates of community screening in Taipei and New Taipei City are from press releases. The mobility data at the village level were provided by the local commercial telecommunication company, Far EasTone Telecommunications. The land use data in 2019 and demographic data including population density and aging index in June 2021 at the village level were obtained from socio-economic database maintained by the Ministry of Interior, Taiwan (https://segis.moi.gov.tw/STAT/Web/Portal/STAT_PortalHome.aspx). The datasets analyzed during the current study are available in the figshare repository [10.6084/m9.figshare.16921120].
