# Supplementary material for: Effectiveness of controlling COVID-19 epidemic by implementing soft lockdown policy and extensive community screening in Taiwan
Source: Sci Rep. 2022 Jul 14;12:12053. doi: 10.1038/s41598-022-16011-x (PMC9282154; doi:10.1038/s41598-022-16011-x)
Supplement: Supplementary file 1 — Supplementary Information 1. [file 41598_2022_16011_MOESM1_ESM.pdf]

Appendix 1. The COVID-19 policy comparison during May-July 2021

Country Name	Stringency Index									Other Policies		
	C1 School closing	C2 Workplace closing	C3 Cancel public events	C4 Restrictions on gatherings	C5 Close public transport	C6 Stay at home	C7 Movement restrictions	C8 International travel	H1 Public information campaigns	H2 Testing policy	H3 Contact tracing	H6 Facial Coverings
Argentina	Require closing	Require closing for some sectors or categories of workers	Require cancelling	Restrictions on gatherings of 10 people or less	Require closing	Require not leaving house with exceptions	Internal movement restrictions in place	Ban on all regions	Coordinated public information campaign	Testing of anyone showing Covid-19 symptoms	Comprehensive contact tracing	Required in all shared/public spaces outside the home
Canada	Require closing	Require closing for all-but-essential workplaces	Require cancelling	Restrictions on gatherings of 10 people or less	Recommend closing	Require not leaving house with exceptions	Internal movement restrictions in place	Ban on all regions	Coordinated public information campaign	Testing of anyone showing Covid-19 symptoms	Limited contact tracing	Required in all shared/public spaces outside the home
Germany	Require closing	Require closing for some sectors or categories of workers	Require cancelling	Restrictions on gatherings of 10 people or less	Recommend closing	Require not leaving house with exceptions	Recommend not to travel between cities	Ban arrivals from some regions	Coordinated public information campaign	Open public testing	Limited contact tracing	Required in some specified shared/public spaces outside the home
India	Require closing	Require closing for all-but-essential workplaces	Require cancelling	Restrictions on gatherings of 10 people or less	Require closing	Require not leaving house with minimal exceptions	Internal movement restrictions in place	Ban arrivals from some regions	Coordinated public information campaign	Testing of anyone showing Covid-19 symptoms	Limited contact tracing	Required outside the home at all times
Taiwan	Require closing	Require closing for some sectors or categories of workers	Require cancelling	Restrictions on gatherings of 10 people or less	No measures	Require not leaving house with exceptions	Recommend not to travel between cities	Ban on all regions	Coordinated public information campaign	Open public testing	Comprehensive contact tracing	Required outside the home at all times
Vietnam	Require closing	Require closing for some sectors or categories of workers	Require cancelling	Restrictions on gatherings of 10 people or less	Require closing	Require not leaving house with exceptions	Internal movement restrictions in place	Ban on all regions	Coordinated public information campaign	Open public testing	Limited contact tracing	Required in all shared/public spaces outside the home
