# Supplementary material for: Effectiveness of controlling COVID-19 epidemic by implementing soft lockdown policy and extensive community screening in Taiwan
Source: Sci Rep. 2022 Jul 14;12:12053. doi: 10.1038/s41598-022-16011-x (PMC9282154; doi:10.1038/s41598-022-16011-x)
Supplement: Supplementary file 2 — Supplementary Information 2. [file 41598_2022_16011_MOESM2_ESM.pdf]

Appendix 2. The procedure of applying spatiotemporal *Gi** statistic in this study.

In this study, the spatiotemporal *Gi** statistic may identify hotspots at a designated week relative to the past few weeks' incidence and can be written as follows:

$$G_i^*(d,t) = \frac{\sum_{t=0}^{m}\sum_{j\in\partial i} w_{ij}(d,t)\cdot x_{jt} - \bar{x}\sum_{t=0}^{m}\sum_{j\in\partial i} w_{ij}(d,t)}{S\sqrt{\frac{n\sum_{t=0}^{m}\sum_{j\in\partial i} w_{ij}^2(d,t) - (\sum_{t=0}^{m}\sum_{j\in\partial i} w_{ij}(d,t))^2}{n-1}}} \tag{1}$$

where $x_j$ is the weekly local COVID-19 incidence of district $j$, $\partial i$ denotes the set of spatial neighbors of district $i$ (including district $i$), $m$ is the designated time lag, $n$ is the number of spatiotemporal neighbors of district $i$ (including location $i$), $\bar{x}$ and $S$ represent the mean and the standard deviation of weekly local COVID-19 incidence of the set of spatiotemporal neighbors of district $i$ (including district $i$), respectively. The spatiotemporal weight for neighbor $j$ from district $i$, $w_{ij}(d, t)$, is defined as follows:

$$w_{ij}(d,t) = (1 - \frac{d_{ij}}{\sum_{j\in\partial i} d_{ij}})^{t+1}, j \in \partial i, \tag{2}$$

where $d_{ij}$ is the distance between locations $i$ and $j$.

Owing to the spatiotemporal structure of the data, neighboring locations exist both in time and space. In this study, polygon contiguity, which means that two spatial units share a common border of non-zero length, is used to define the neighborhood size in space. To define temporal neighbors, time-lagged correlations, which describe how

similar the time series is with itself, are calculated and tested until the first statistically insignificant result appears with a significance level of 0.05. The result of the time lag selection here was eight weeks.
