# Supplementary material for: Effectiveness of controlling COVID-19 epidemic by implementing soft lockdown policy and extensive community screening in Taiwan
Source: Sci Rep. 2022 Jul 14;12:12053. doi: 10.1038/s41598-022-16011-x (PMC9282154; doi:10.1038/s41598-022-16011-x)
Supplement: Supplementary file 3 — Supplementary Information 3. [file 41598_2022_16011_MOESM3_ESM.pdf]

Appendix 3. The procedure of computing degree-centrality from mobility data.

For each village i, the telecommunication company will compute how many people spend more than 15 min in village j. Then, Pij is the percentage of the time and mobile users stayed in village i and visited village j for some time in minutes. For each village i, the summation of Pij is close to one. For some visiting fewer than 15 min, it will be lost in the computation process. The Pij for each village was computed using four age groups. To protect the privacy, if the village with the number of mobile phone users fewer than five, the telecommunication company treated it as "*" symbol. When we analyzed the data, we replaced the case with three persons for computation.

When we computed the weighted degree centrality, we first removed those self-linked pairs (Pii) that did not move among villages. We then computed weighted in-degree (Wij-in) and out-degree centrality (Wij-out) for each age group using the igraph package (https://igraph.org/r/) in R software. For each Wij, we compute the Pij times the mobile phone users of FarEasTone in the corresponding villages.
